# Investigation on Life Satisfaction of Rural-to-Urban Migrant Workers in China: A Moderated Mediation Model

**DOI:** 10.3390/ijerph17072454

**Published:** 2020-04-03

**Authors:** Qian Liu, Haimin Pan

**Affiliations:** 1 School of Public Administration, Hunan Normal University, Changsha 410081, China; liuqian67520@126.com; 2 School of Sociology and Anthropology, Xiamen University, Xiamen 361005, China; 3 Department of Sociology, Zhejiang University, Hangzhou 310058, China

**Keywords:** subjective relative deprivation, linking social capital, life satisfaction, friendship network size, rural-to-urban migrant workers

## Abstract

Given the large number of internal migrant workers in China and their contribution to the development of the society and country, their life satisfaction somewhat signifies the success of their integration into the new environment. This study is to explore the influence of subjective relative deprivation on life satisfaction among rural-to-urban migrant workers in China. Additionally, linking social capital was used as a mediator and friendship network size as a moderator. This study was designed as cross-sectional and 2442 rural-urban migrants in both Xiamen (*N* = 1197) and Changsha (*N* = 1245) were recruited by using a multi-stage stratified probability proportional to size sampling method. The results showed a significantly negative mediating effect of linking social capital with a negative association between subjective relative deprivation and life satisfaction. Moreover, the number of friends moderated the effect of linking social capital on life satisfaction. This study confirmed a moderated mediation model and highlighted the importance of linking social capital and subjective relative deprivation in deciding life satisfaction of Chinese rural-to-urban migrant workers. Polities and purposeful action for enhancing social integration (i.e., interaction with community members and taking part in social and political activities) are advised to build a well-off society in China.

## 1. Introduction

China has witnessed tremendous rural-to-urban migration due to social and economic development since 1978 [1], with nearly 288.36 million rural migrants moving to the cities in 2018 [2]. Migration is a complex process of adaption to the host society, which may stir up disruptive experiences [3]. Research on international immigration has unmasked worse mental conditions among this group of people when compared to local ones [4,5], and this is the same case with internal migrants in China [6]. Since 1958, China has implemented the system of residential registration (hukou) and each citizen can be categorized as having an “urban” or “rural” belonging [5]. The public resources and social welfare are distributed according to hukou status [7]. That is, the welfare services of a city are only provided to those urban residents with a local hukou [8]. As a result, rural-to-urban migrants do not have access to full citizenship rights and are deprived of various social, economic, and political rights [9,10,11]. They are treated as “second-class citizens,” suffering from discriminations in employment, housing, education and health care in cities [12]. Such social stigma and discrimination, for example, predict lower levels of quality of life and happiness than dwellers with a local hukou [13,14]. To be noted, migration is not a linear routine over time because of multiple elements [15]. These multiple elements include risk factors (e.g., language barriers and cultural shock) and protective factors (e.g., social support and resilience), and matter in deciding a migrant’s life quality and mental health [16]. Therefore, it is valuable to update the explanations for the mechanisms of risk and protective factor effects on migration outcomes. This study centered on subjective relative deprivation, linking social capital and friendship network size, and investigated their roles in deciding internal migrants’ life satisfaction.

## 2. Literature Review

### 2.1. Subjective Relative Deprivation and Mental Health

Relative deprivation was originally defined as “a state of observable and demonstrable disadvantage, relative to the local community or the wider society or nation to which an individual, family or group belongs” p. 125 [17]. This disadvantage can be observed objectively and additionally can be the subjective perception of a person when he/she makes a comparison with their reference groups (e.g., neighbors, the local residents, and the same cohort of migrants) in terms of life resources. In this sense, subjective relative deprivation reflects the feelings of resentment, dissatisfaction, and anger on the inequality between real situations and the standard levels that people expect [18]. Those inferior feelings have detrimental consequences, such as unhappiness or low levels of subjective well-being [18,19]. Those moving from countryside regions to cities tend to experience a sense of relative deprivation due to social exclusion. It is said that internal migrants in China reside in the receiving communities temporarily and often move between cities and communities circularly without an urban hukou [20]. An urban hukou is potentially linked to some forms of social resources. Moreover, migrants may suffer from other unobservable disparities, like discrimination and stigma, that lead to poorer mental health among the internal migrant population [14]. Economic inequality is a factor that serves as one of the main reasons for the decision of migration intra-personally, as well as for psychological outcomes that migrants experience when adapting to the host cities. It was reported that feeling economically deprived relative to others of interest weighs heavily in migrants’ subjective well-being [19]. Together, relative deprivation in economic conditions can mirror the mental health of internal migrants in China. Such investigation is not adequate and deserves more efforts [20].

### 2.2. Linking Social Capital and Mental Health

Social capital is a concept containing multifaceted dimensions [21]. Among the variety of approaches in conceptualization and measurement of social capital, Putnam and Goss conceptualized social capital as two sub-concepts bonding social capital and bridging social capital, which denote the value and networks shared among people of homogeneous and heterogeneous groups, respectively [22]. Afterwards, Szreter and Woolcock put forward another sub-concept of linking social capital and defined it “as norms of respect and networks of trusting relationships between people who are interacting across explicit, formal or institutionalized power or authority gradients in society” (p. 655) [23]. Since then, these three concepts have been tied up as an alternative framework to represent the broader concept of social capital. In a study with a sample of 2985 Chinese people, linking social capital measured by political participation and political efficacy was proven to be influential on the mental health of rural, urban, and global China, while bonding social capital was significantly positively related to physical and general health in rural and global Chinese people. Furthermore, bridging social capital was significantly negatively related to mental health in urban and global China [24]. The results of that research revealed that linking social capital has unique and higher validity in predicting mental health among the Chinese population. Unlike a study in a Western society that used voting as an important indicator of linking social capital [25], Jiang and Wang again verified the significant association between linking social capital and mental disorders among Chinese adults by using political trust, political efficacy, political participation and membership in the Chinese Communist Party (CCP) as multifarious aspects of linking social capital [26]. This distinction reflects social and cultural differences between Chinese and Western settings, with the former, for example, being highly faithful in and following orders whenever and wherever and labeled as a power-oriented society [26]. Overall, people with higher linking social capital usually have deeper political engagement and thus have more trust in government authority and are more likely to be informed by health promotion for healthier behaviors [24]. Regarding the migrant population, their unauthorized condition could be settled with high social capital to gain access to resources for their health conditions [27]. Research on how linking social capital functions among internal migrants regarding their mental health is, to our best knowledge, unavailable but valuable.

### 2.3. Friendship as a Moderator

Social interaction with peers influences the well-being of people, including internal migrant children [28]. Good friendship is beneficial in offering social support for dealing with psychological distress and solving problems [29]. However, as an internal migrant population, they tend to spend less time in effortful activities (e.g., leisure activities, meeting people) as compared to local counterparts, and thus lack the opportunities for making a new, high quality friend out of the people around [30]. Therefore, in spite of the benefits a friendship network may provide, internal migrants still face a hardship in keeping a good network with people of interest. It is referable that the quality of friendship matters the most for internal migrants. Within the internal migrant workers, these who have more friends and acquaintances (larger friendship network size) end with better mental health, with the network effect channeled through the reduction of migrants’ anxiety and by boosting their confidence [6]. Additionally, regarding the origins of political attitudes and behaviors, friends, apart from families, in effect, weigh a lot in formulating adults’ political attitudes and behaviors. A good friendship network could help build a social environment where a person will learn about the appropriate attitudes and behaviors for becoming integrated into the society and gain social capital and political participations [31]. Due to the fact that shrinking social capital may reduce the opportunities for rural-urban migrant workers to make friends in host cities [30], the effect of linking social capital on mental health among internal migrants could correspondingly vary. Thus, a friendship network could be a potential moderator in such a relationship.

### 2.4. Subjective Relative Deprivation and Linking Social Capital

Subjective relative deprivation is not only highly influential in the mental health of the internal migrants in China, but also in the level of linking social capital. According to rational choice theory, political trust stems from people’s evaluation of the government performance [32]. Income inequality is an important index for government economic performances [33]. The more unfair the income distribution is, the less likely that the public will trust the government [34,35]. The subjective relative deprivation in terms of economic status can reflect the perception of income distributive fairness. Accordingly, subjective relative deprivation has been negatively associated with political trust [36].

Furthermore, in view of the statement of Jin that rural-to-urban migrants were typically situated in the lower social hierarchy and the special rural-urban dual system in China [20], it is conceivable that those migrants with higher levels of subjective relative deprivation may possess fewer opportunities for taking part in political activities and their trust in the government may be alarming. In actuality for internal migrants in China, institutional exclusion and discrimination or hukou identity is impactful in eroding migrants’ sense of social fairness and thus decreases their trust in government and political efficacy [17]. It is noted that social integration could serve as a catalyst for economic incorporation of migrants into the host country’s fabric [37]; it has been proposed that higher subjective relative deprivation in terms of economic status among migrants reflects worse levels of adaption to the receiving communities, and therefore a more negative impression on government performance. This notion accords with the claims that economic status is an important criterion used to evaluate how well the local government performs [17]. Therefore, it is inferable that subjective relative deprivation in terms of economic status has potential in predicting linking social capital as indicated by political confidence.

### 2.5. Internal Migrants’ Life Satisfaction

**Figure 1:**
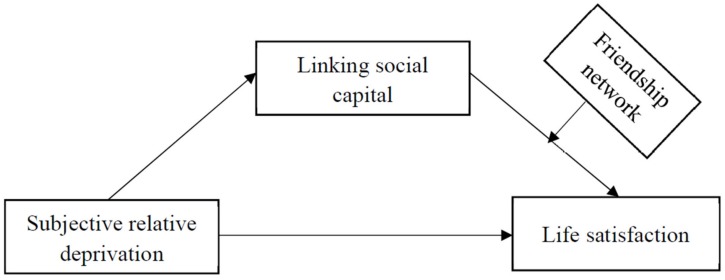
The conceptual model including subjective relative deprivation, linking social capital, life satisfaction and friendship network.

Life satisfaction is one of the important themes reflecting the consequence of integration into the host society for migrants that has received attention in the field of migration study [5,38]. It endorses a positive attitude toward overall life [39]. However, evidence on the role of linking social capital in developing life satisfaction among Chinse internal migrants is pending exploration. As for migrants, when they leave the places they are familiar with for a new environment, they need to adjust to involvement in the unfamiliar circumstances while meanwhile facing the issue of losing familiar social networks and social environments. Rural migrants in China are always viewed as “guest workers” in Chinese cities and are not entitled to the social services and welfare mainly received by the local urban counterparts [6]. This institutionalized discrimination due to the special hukou registration system aggravates the burden on the rural migrants as compared to those in most cases. In effect, rural people choose to leave their hometowns to urban societies for a better quality of life, though migration is a stressful life experience. Good life satisfaction, on one hand, serves as a protective factor for the migration outcomes [40], but on the other hand, it is the ultimate aim of the migration that the migrants pursue. Life satisfaction has a wide scope of meaning in describing the life status of a person. It not only indicates an overall evaluation of one’s life especially in the aspect of interpersonal relationships, but also shows concerns with one’s relative standard of living or material comforts [41]. That is why economic achievement cannot stably and solely predict the levels of life satisfaction among Chinese migrant workers [41,42]. In particular, interpersonal relationships allow for the interchange of financial and human resources between people through which one’s well-being and health is improved [43]. Social capital is one form of culmination of mobility through which resources flow. Thus, this study broadens the horizon in explaining the results of life satisfaction among Chinese rural-to-urban migrants when taking economic and social factors into consideration together. In sum, the information above can be synthesized and is visualized in Figure 1.

Hypotheses of relevance were proposed below:

**Hypothesis** **1.** *There is a negative relationship between subjective relative deprivation and life satisfaction among rural-to-urban migrant workers.*

**Hypothesis** **2.** *Linking social capital serves as a mediator in the relationship subjective relative deprivation and life satisfaction among rural-to-urban migrant workers.*

**Hypothesis** **3.** *Friendship network size moderates the relationship between linking social capital and life satisfaction among rural-to-urban migrant workers.*

## 3. Methods

### 3.1. Subjects and Procedure

This study was conducted in Xiamen and Changsha in 2016, both of China’s biggest cities. Xiamen City is a southeastern coastal city administered under the Fujian province. It was one of China’s earliest Special Economic Zones in the 1980s and is one of the key receiving locations for rural-urban migrants. Changsha City is the economic and cultural center in Hunan Province or even in the whole of Midwest China, a major destination place for rural migrants in the central region of China. With the highest proportion of migrants, Xiamen and Changsha serve as excellent examples of rural-urban migrant receiving areas in China.

By employing a multi-stage stratified probability proportional to size (PPS) sampling design, the researchers first selected 6 districts across the Xiamen/Changsha City and 4–6 communities within each district. For each community, 40–45 rural-urban migrants were randomly chosen from the floating population’s community rosters. Thirty RMB (4.28 USD) were given as an incentive to each subject, being twice as much as the average hourly wage of the two cities involved. It took about 40 min on average for subjects who were rural-urban migrants above age 16 with agricultural hukou to complete the questionnaire in community centers. To ensure a precise comprehension of the questions, all the subjects completed the questionnaire orally through an ask-and-answer procedure that was assisted by an interviewer. This research obtained ethical approval from the Hunan Normal University Research Ethics Committee. Overall, the survey covered approximately 2442 rural-urban migrants in both Xiamen (*N* = 1197) and Changsha (*N* = 1245).

### 3.2. Measures

The questionnaire contained the following sociodemographic information of the subjects: age, gender, marital status, education, income, occupation, immigratory place, and immigration distance.

Dependent variables: Life satisfaction was seen as indicative of subjective well-being. A single question about “How do you feel about your current life?” was rated by the subjects on a 5-point Likert scale ranging from 1 (very dissatisfied) to 5 (very satisfied). A higher score indicated a higher sense of life satisfaction.

Independent variables: The presence of a reference group was necessary for measuring the subjective relative deprivation. Previous studies suggested that members of one’s reference group were typically selected on the basis of either similarity or geographic proximity [44]. Accordingly, we selected both citizens and migrants around the subjects as the reference group. Subjective relative deprivation was measured with two dimensions: comparison with the citizens (e.g., “Is your family economic status currently better than the citizens around you?”) and comparison with the rural-urban migrants (e.g., “Is your family economic status currently better than the rural-urban migrants around you?”). The subjects responded to these two questions with a rating from 1 (better) to 3 (worse). The scores of the items were summed up and higher scores represented higher levels of subjective relative deprivation.

Mediator: Linking social capital was measured with self-designed items in terms of political trust, which were adopted from the Center for Political Studies election study (The survey was based on The University of Michigan Survey Research Center election studies of a national cross-section of eligible studies, and is now conducted by the Center for Political Studie, CPS) [45]. The items were, “Do you think the governments in Xiamen/Changsha always do what is right?”, “Do you think that the governments in Xiamen/Changsha always protect the interests of the people?”, “Do you think that the government administrative expenses used by the government officials are always reasonable?”, “How many officials do you think are competent in running the Xiamen/Changsha government?”, “How many officials in the Xiamen/Changsha government do you think are honest and reliable?”. The subjects indicated their responses on 4-point scale ranging from 0 (never/none) to 3 (always/all). The scores of the total five items were computed and a higher score indicated higher levels of linking social capital. Cronbach’s alpha coefficient for the linking social capital scale was 0.863. Exploratory factor analysis showed that the five items were loaded on one dimension together, explaining 65.35% of the variance. The values of factor loadings ranged from 0.769 to 0.829. These results showed that the measure of linking social capital in this study had good psychometric properties.

Moderator: Friendship network size was measured with a self-reported item. A question “How many friends do you have in the city?” was available for the subjects to report about the specific number of their friends.

### 3.3. Statistical Analysis

Age, gender, marital status, education, income, occupation, immigratory place, and immigration distance were used as covariates in this study. Path analysis was used to explore the relationships among life satisfaction, subjective relative deprivation, linking social capital, and friendship network size. In order to retrieve as much information as possible from observations containing missing values, we used the maximum likelihood with missing values (MLMV) method to estimate structural equations.

The bootstrapping procedure could reduce the likelihood of Type I errors [46]. As the value of a*b did not meet the requirement of normal distribution, we ran the bootstrapping procedure 500 times additionally to produce the bias-corrected confidence intervals (CI) for the inference test for the indirect effects. Specifically, this procedure involved 500 instances of resampling with replacement from which a sampling distribution of the indirect effect was built and could be used to construct CI. CI that excluded zero indicated significant indirect effects.

**Figure 2:**
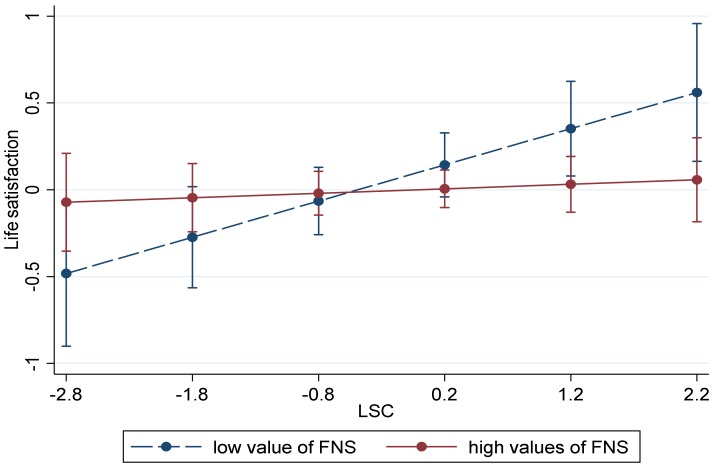
The association between linking social capital and life satisfaction with friendship network size as a moderator. Note: LSC = linking social capital, FNS = friendship network size.

Standardized coefficients were reported, and z scores were used for product terms in the SEM model. Moderated mediation analysis was a second-stage moderation model [47]. That is, friendship network size would moderate the relationship between linking social capital and life satisfaction, resulting in a stronger indirect effect when friendship network size was low than when it was high. We graphed the interaction terms and presented the slopes of linking social capital on life satisfaction at high and low values of the friendship network size in Figure 2. The high and low friendship network size were defined as one standard deviation above and below the mean, respectively.

In order to provide a formal test for the moderated mediation effect, we examined the index of moderated mediation [48]. The index was an interval estimate of the parameter of a function linking the indirect effect to values of a moderator. The two conditional indirect effects defined by the moderator’s various values were statistically different if the index was significance.

## 4. Results

The survey included 2442 rural-urban migrants with an average age of 34.54 ± 10.37 (range: 16–76) years. Subjects’ sociodemographic and migratory information are presented in Table 1.

The correlations of the variables are showed in Table 2. Subjective relative deprivation (r_life satisfaction_ = −0.29, *p*_s_ < 0.05) and linking social capital (r_life satisfaction_ = 0.09, *p*_s_ < 0.05) were significantly associated with life satisfaction, but friendship network size (r_life satisfaction_ = 0.02, *p*_s_ = ns) wasn’t significantly associated with life satisfaction. In addition, subjective relative deprivation was positively associated with linking social capital (r = 0.05 *, *p*_s_ < 0.05).

**Figure 3:**
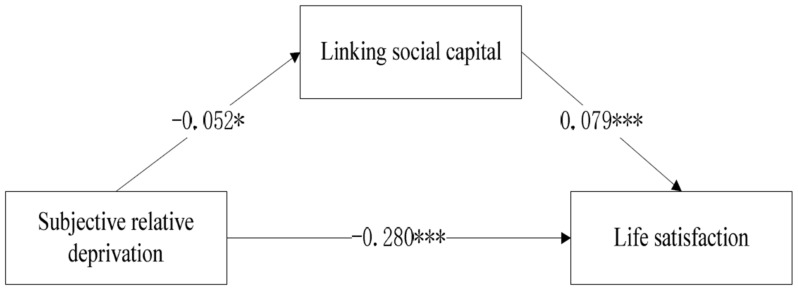
A path model of how subjective relative deprivation influences life satisfaction. * *p* ≤ 0.05; *** *p* ≤ 0.001.

Table 3 presents subjective relative deprivation’s indirect effects on life satisfaction. The result showed that the fit indices of the model met the recommended criteria, suggesting a good model fit with statistical significance: log likelihood (−39,318.901) and coefficient of determination (CD = 0.151). The direct effects of subjective relative deprivation on linking social capital and life satisfaction were −0.052 (*p* < 0.005), and −0.280 (*p* < 0.0001), respectively; the direct effect of linking social capital on life satisfaction was 0.079 (*p* < 0.0001). The indirect effect of subjective relative deprivation was −0.004 (*p* < 0.005). The overall effect of subjective relative deprivation on life satisfaction was −0.284 (*p* < 0.0001); and that of linking social capital on life satisfaction was 0.079 (*p* < 0.0001). The important values of the model are shown in Figure 3.

**Figure 4:**
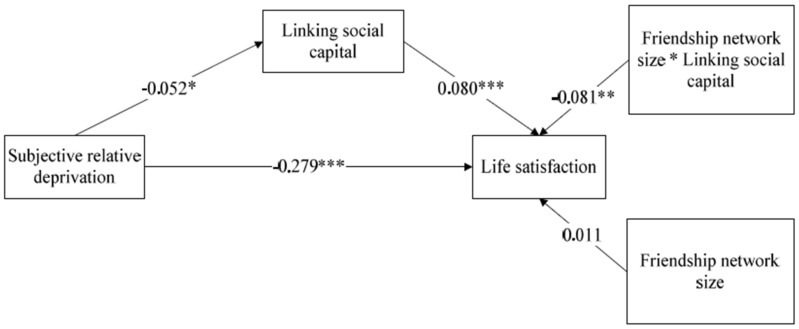
A path model of how subjective relative deprivation influences life satisfaction with friendship network size as a moderator. * *p* ≤ 0.05; ** *p* ≤ 0.01; *** *p* ≤ 0.001.

Table 4 depicts the findings of the moderated mediation analysis. The result illustrated that the fit indices of the model met the recommended criteria, suggesting a good model fit with statistical significance: log likelihood (−45,469.004) and coefficient of determination (CD = 0.156). The interaction term friendship network size × linking social capital was significant in the moderated mediation models (Table 4). The comparison between the mediation model and moderated mediation model captured an additional considerable variance explained in life satisfaction (△Log likelihood = 6150.103, △CD = 0.005), which indicated a potential occurrence of a moderated effect in the mediation model. The direct effects of subjective relative deprivation on linking social capital and life satisfaction were −0.052 (*p* < 0.005), and −0.279 (*p* < 0.0001), respectively; the direct effect of linking social capital on life satisfaction was 0.080 (*p* < 0.0001); the direct effect of friendship network size on life satisfaction was 0.011 (*p* > 0.05) and the direct effect of friendship network size × linking social capital was −0.081 (*p* < 0.01). The indirect effect of subjective relative deprivation was −0.004 (*p* < 0.005). The overall effect of subjective relative deprivation on life satisfaction was −0.283 (*p* < 0.0001) and that of linking social capital on life satisfaction was 0.080 (*p* < 0.0001); that of friendship network size on life satisfaction was 0.011 (*p* > 0.05), and that of friendship network size × linking social capital was −0.081 (*p* < 0.01). The important values of the model are shown in Figure 4.

Detailed information on the moderating role of the friendship network size in the indirect effects is visible in Figure 2. Compared with individuals with high friendship network size, the associations between linking social capital and life satisfaction were weaker than individuals with a low friendship network size. Additionally, the index of moderated mediation was significant (index = 1.4%,95% CI [−0.008, −0.00005]), which indicated that the indirect effect that subjective relative deprivation had on life satisfaction through linking social capital varied depending on the value of personal friendship network size.

To illustrate the presence of moderated mediation, we reported the indirect effect at various levels of the moderator in Table 5 at 1 SD below the mean, the mean, and 1SD above the mean. For individuals with low levels of friendship network size, the indirect effect was significant (−0.008, 95% CI [−0.0191, −0.0018]). For individuals with mean levels of friendship network size, the indirect effect was significant (−0.004, 95% CI [−0.0109, −0.0010]). For individuals with high levels of friendship network size, the indirect effect became nonsignificant (−0.0001, 95% CI [−0.0042, 0.0031]). These results indicate that subjective relative deprivation’s indirect effect on relocation life satisfaction through linking social capital is contingent on personal friendship network size, such that higher levels of personal friendship network size decrease the indirect effect’s magnitude.

## 5. Discussion

This study explored the mediation mechanism between subjective relative deprivation, linking social capital, and life satisfaction when linking social capital acted as a mediator. The moderator of friendship network size was included in the mediation model, and a moderated mediation model was examined. It turned out that subjective relative deprivation did lower the perceived life satisfaction among rural-to-urban migrant workers (Hypothesis 1), and the association between subjective relative deprivation and life satisfaction was partially dependent on the levels of linking social capital (Hypothesis 2). Moreover, the effect of linking social capital on life satisfaction was weakened when friendship network size became broader (Hypothesis 3). These results contribute to the theoretical and practical implications in terms of how to enhance the life satisfaction of rural-to-urban migrant workers by reducing their subjective relative deprivation and increasing the linking social capital instead. Subjective relative deprivation may reflect the status of lower political hierarchy and less opportunities for undertaking political activities as compared to their peers (local citizens and migrants around the subjects). This feeling of inferiority could breed the internal migrants’ feeling of unfairness and distrust towards the government [17]. This also accords with the statement of Li and Wu that citizens’ political trust, to a large extent, is determined by their material benefits received [49]. It is also true that economic deprivation relative to others of interest could exacerbate subjective well-being problems among internal migrants, which was consistent with the statement of the study by Liu et al. [19]. This study added evidence to the notion that higher levels of linking social capital (trust in the government) that rural-to-urban migrants workers possess would secure their life satisfaction. The improved life satisfaction may result from the accompanying social and political resources that linking social capital could provide for the health-related outcomes [27], particularly for Chinese people [24]. The partial mediating role of linking social capital in the relationship between subjective relative deprivation and life satisfaction, as well as subjective relative deprivation, in effect mirror the political and economic pathways in deciding the health conditions of internal migrants in China.

This study also found that friendship network size mattered for life satisfaction among rural-to-urban migrant workers. Contrary to the study by Meng and Xue [6], this research did not support the positive effect of having more friends and acquaintances on mental health among internal migrant workers. The size of the friendship network itself had no significant influences on life satisfaction, while the association between linking social capital and life satisfaction became weaker when the number of friends and acquaintances increased. According to Zhao and Hu who revealed that citizens who were younger, more highly educated and well-paid have a lower probability of trust in government in China [50], it is seen that people with higher social resources could decrease their dependence on the government for a better life. Given the phenomenon of reliance on materials impacting political trust in Chinese people [49], it is inferable that the friendship network undertakes part of the role of bringing benefits to the people involved, which leads to the apportioned effect of friendship network size in life satisfaction from the linking social capital. Although the direct effect of friendship network size was statistically invalid, it can work together with linking social capital in determining life satisfaction among internal migrant workers.

Implications of this study merit focused attention. This study shed light on the mechanism of life satisfaction among rural-to-urban migrant workers in China, including economic (subjective relative deprivation), political (linking social capital), and social aspects (friendship network size). There have been hundreds of articles investigating the relationship between social capital and health outcomes, and this subject continues to be exposed in the literature [51]. However, linking social capital, compared to other dimensions of social capital, has been inadequately studied. Although rural-to-urban migrant workers choose to migrate mainly for economic purposes, their life cannot go well when it is secluded from political realms. Regarding the fact that policy benefits could affect people’s political attitudes [52], the policies that incline to the interests of internal migrant workers are highly recommended. In addition, measures aiming to increase social integration so internal migrant workers own into their living circumstance are also promising. Social integration could actually catalyze the economic incorporation of migrants into the host country’s fabric [37]. In this sense, subjective relative deprivation through comparison with familiar others would be attenuated, and thus life satisfaction could be enhanced. Joining the interactions of community members [53] and engaging in a variety of community and civic activities [54] are effective approaches for increasing social integration. To build a harmonious and well-off society, as the government has advocated for many years, the benefits of internal migrant workers, including economic, political, and social interests, should also receive great importance.

Many limitations need to be acknowledged. First, the cross-sectional design of this study limits more accurate casual explanations for the observation. Second, Young et al. found that the effect of peer support varied with age and that older adolescents benefited the most from high peer support [55]. In this study, the mean age of the sample was beyond the age ranges of older adolescents. Thus, further efforts could pay attention to other age cohorts of rural-to-urban migrant workers since the effects explored in this study could vary because of age diversity, especially the effect of the friendship network. Third, self-reported measures could induce response bias, for example, acquiescence [42], which in turn may result in overestimation of the association between variables in this study. Last but not least, only political trust was used as indicative of linking social capital, and other dimensions such as political efficacy, political participation and membership in the Chinese Communist Party (CCP) could be taken into account in further studies. Similarly, the quality of friendship, not the number, could be another precious angle for detecting the effect of the friendship network. According to Martin and Huebner, good friendship endorses social support and helps to deal with psychological distress and helps to solve problems [29]. Thus, the quality of the friendship network might have an obvious direct effect on life satisfaction, which needs further evidence.

## 6. Conclusions

This study emphasized the importance of linking social capital and subjective relative deprivation on internal migrants’ life satisfaction. The effect of the friendship network size was also considered. The hypotheses in this study were verified in the results. That is, subjective relative deprivation had a direct negative impact on life satisfaction and indirectly affected life satisfaction through linking social capital. Such an indirect effect is dependent on personal friendship network size. The partial mediating role of linking social capital and the direct effect of subjective relative deprivation, as well as marked moderated effect of friendship network size, essential policies and actions, can increase the social integration of rural-to-urban migrant workers in host cities and help them in achieving a satisfied life. Future research would be feasible and valuable if many interventions are attempted for improving the life satisfaction of internal migrants.

## Figures and Tables

**Table 1:** Personal information about the subjects.

Variable	Mean ± SD (Range) *N* (%)	Number of Cases
Age	34.54 ± 10.37 (16–76)	2435
Gender		
Male	1394 (57.08%)	2442
Female	1048 (42.92%)	
Marital status		
With a spouse	1793 (73.48%)	2440
Single (unmarried/divorced/widowed)	647 (26.52%)	
Education		
Primary education or below	277 (11.35%)	2441
Junior high education	1001 (41.01%)	
Senior high or technical secondary education	781 (32.00%)	
tertiary education and above	382 (15.65%)	
Monthly income (RMB)	3866.053 ± 1861.695(1500–9000)	2359
Occupation		
Employed	1706 (77.97%)	2188
Self-employed	482 (22.03%)	
Duration of stay	8.40 ± 7.46(1–63)	2436
Immigration distance		
Inter-provincial migration	911 (37.31%)	2442
Inner-provincial migration	1531 (62.69%)	
Immigratory place		
Changsha	1245 (50.98%)	2442
Xiamen	1197 (49.02%)	

SD = standard deviation, 1 USD = 7.011RMB.

**Table 2:** Descriptive statistics and bivariate correlations of the study variables.

Variable	Mean ± SD	1	2	3	4
1. Subjective relative deprivation	4.36 ± 0.89	1	−0.05 *	−0.07 *	−0.29 *
2. Linking social capital	1.59 ± 0.57		1	0.05 *	0.09 *
3. Friendship network size	34.95 ± 31.38			1	0.02
4. Life satisfaction	3.59 ± 0.81				1

SD = standard deviation, * *p* < 0.05.

**Table 3:** The effects of subjective relative deprivation on life satisfaction through linking social capital.

Variable	Linking Social Capital	Life Satisfaction		
		Total Effects	Direct Effects	Indirect Effects
Subjective relative deprivation	−0.052 *	−0.284 ***	−0.280 ***	−0.004 *[−0.008, −0.00005]
Linking social capital	—	0.079 ***	0.079 ***	—
Age	0.171 ***	0.084 **	0.071 **	0.013 **
Gender	0.010	−0.049 *	−0.050 *	0.001
Marital status	−0.015	−0.004	−0.002	−0.001
Education	0.035	0.051 *	0.048 *	0.003
Income	−0.019	0.010	0.011	−0.002
Occupation	−0.004	0.018	0.017	−0.0003
Duration of stay	−0.028	0.044	0.046 *	−0.002
Origin areas	−0.002	−0.008	−0.008	−0.0001
Immigratory place	0.222 ***	−0.0132	−0.031	0.017 **

Unstandardized coefficients are presented. CI = confidence interval. Bootstrap = 500. * *p*< 0.05, ** *p* < 0.01, *** *p* < 0.001, —, no link specified in the model.

**Table 4:** The effects of subjective relative deprivation on life satisfaction through linking social capital with friendship network size as a moderator.

Variable	Linking Social Capital	Life Satisfaction		
		Total Effects	Direct Effects	Indirect Effects
Subjective relative deprivation	−0.052 *	−0.283 ***	−0.279 ***	−0.004 * [−0.008, −0.0001]
Linking social capital	—	0.080 ***	0.080 ***	—
Friendship network size	—	0.011	0.011	—
Linking social capital × Friendship network size	—	−0.081 **	−0.081 **	—
Age	0.171 ***	0.083 ***	0.070 **	0.014 **
Gender	0.010	−0.052 *	−0.053 **	0.001
Marital status	−0.015	−0.003	−0.001	−0.001
Education	0.035	0.051 *	0.048 *	0.003
Income	−0.019	0.007	0.009	−0.002
Occupation	−0.004	0.019	0.019	−0.0003
Duration of stay	−0.028	0.041	0.044 *	−0.002
Origin areas	−0.002	−0.006	−0.006	−0.0001
Immigratory place	0.222 ***	−0.0132	−0.031	0.018 ***

Unstandardized coefficients are presented. CI = confidence interval. Bootstrap = 500, * *p* < 0.05, ** *p* < 0.01, *** *p* < 0.001, —, no link specified in the model.

**Table 5:** The conditional indirect effects.

Variable	Coef.	BootSE	z	*p* > |z|	BC [95% CI]	
Low Friendship network size	−0.008	0.004	−20.08	0.037	−0.0191	−0.0018
Average Friendship network size	−0.004	0.002	−10.94	0.052	−0.0109	−0.0010
High Friendship network size	−0.0001	0.002	−0.07	0.946	−0.0042	0.0031

BC = bias-corrected confidence interval. CI = confidence interval. Bootstrap = 500.
